# High-Throughput Sequencing of Oral Microbiota in *Candida* Carriage Sjögren’s Syndrome Patients: A Pilot Cross-Sectional Study

**DOI:** 10.3390/jcm12041559

**Published:** 2023-02-16

**Authors:** Haixia Xing, Hongwei Liu, Jie Pan

**Affiliations:** 1 Department of General Dentistry, National Center for Stomatology, National Clinical Research Center for Oral Diseases, National Engineering Research Center of Oral Biomaterials and Digital Medical Devices, Peking University School and Hospital of Stomatology, Beijing 100081, China; 2 Department of Oral Medicine, National Center for Stomatology, National Clinical Research Center for Oral Diseases, National Engineering Research Center of Oral Biomaterials and Digital Medical Devices, Peking University School and Hospital of Stomatology, Beijing 100081, China

**Keywords:** Sjögren’s syndrome, high-throughput nucleotide sequencing, dental caries, saliva microbiota

## Abstract

Background: This study sought to characterize the saliva microbiota of *Candida* carriage Sjögren’s syndrome (SS) patients compared to oral candidiasis and healthy patients by high-throughput sequencing. Methods: Fifteen patients were included, with five *Candida* carriage SS patients (decayed, missing, and filled teeth (DMFT) score 22), five oral candidiasis patients (DMFT score 17), and five caries active healthy patients (DMFT score 14). Bacterial 16S rRNA was extracted from rinsed whole saliva. PCR amplification generated DNA amplicons of the V3–V4 hypervariable region, which were sequenced on an Illumina HiSeq 2500 sequencing platform and compared and aligned to the SILVA database. Taxonomy abundance and community structure diversity was analyzed using Mothur software v1.40.0. Results: A total of 1016/1298/1085 operational taxonomic units (OTUs) were obtained from SS patients/oral candidiasis patient/healthy patients. *Treponema*, *Lactobacillus*, *Streptococcus*, *Selenomonas*, and *Veillonella* were the primary genera in the three groups. The most abundant significantly mutative taxonomy (OTU001) was *Veillonella parvula*. Microbial diversity (alpha diversity and beta diversity) was significantly increased in SS patients. ANOSIM analyses revealed significantly different microbial compositional heterogeneity in SS patients compared to oral candidiasis and healthy patients. Conclusion: Microbial dysbiosis differs significantly in SS patients independent of oral *Candida* carriage and DMFT.

## 1. Introduction

Sjögren’s syndrome (SS) is a systemic autoimmune exocrinopathy characterized by mouth and eye dryness, fatigue, joint pain, and other symptoms [1]. As an effect of pathology, the oral manifestations of SS include xerostomia and hyposalivation, dental caries, oral candidiasis, dental erosion and/or abrasion, and others [2]. In 2015, untreated dental caries affected an estimated 2.6 billion adults globally. That year, oral conditions contributed to the declined health in 35 of 39 categories of cancer [3]. Among all adults with dental caries, patients with SS showed a significantly higher dental caries rate compared to non-SS patients [4]. SS patients are also more likely to have had teeth extractions [5] and have a higher rate of decayed, missing, and filled teeth (DMFT) [6]. 

The development of dental caries depends on the balance of pathological and protective factors. Pathological factors include acidogenic bacteria, inhibition of salivary function, and frequency of ingestion of carbohydrates [7]. Hyposalivation (defined as a whole salivary flow rate <0.1 mL/min without stimulation) is an important influence during the development of dental caries in SS [8]. Hyposalivation might delay the clearance of sugar and acid from the dental surface. The lack of buffers influences the survival and growth of oral bacteria and fungi [9], leading to dental caries, erosion, candidiasis, etc. [10]. For SS patients with hyposalivation, antibacterial methods might be a necessary intervention to manage dental caries [11]. 

Changes in the oral microbiota can involve many pathological and non-pathological aspects within the oral cavity [12]. Knowledge of the oral microbiota is important for the management of dental caries. However, whether the oral microbiota in SS patients differ from healthy individuals is unclear. Generally speaking, quantitative and qualitative changes in saliva might lead to specific changes in the salivary microbial composition, with increasing cariogenic colonization by *Streptococcus mutans* and *Lactobacillus* [13]. Colonization by cariogenic and acidophilic microorganisms in the microbiota elevates the risk of caries [4]. Microbial dysbiosis has been described in SS patients with normal salivary flow rates [14]. However, a comparable salivary microbiota was found in hyposalivation patients, compared with control patients having normal saliva flow rates and comparable DMFT [15,16]. Many other factors might contribute to the salivary microbiota in SS patients, making comparison of the findings of studies difficult [17]. 

Among these factors, oral health status might be influential, including the rate of dental caries and/or DMFT. Even without medical conditions affecting saliva, the mean prevalence of dental caries reported in one study was 140% higher in hyposalivation patients than the healthy control group [18]. Other authors reported that with comparable DMFT, the salivary microbiota in the SS patients was not significantly different from that of healthy controls, with the most abundant genera in both groups being *Veillonella, Streptococcus, Prevotella,* and *Haemophilus* [19]. With higher DMFT in SS patients, the relative abundance of *Veillonella* was also reportedly higher than healthy controls, while *Neisseria, Actinomyces, Haemophilus, Rothia, Porphyromonas*, and *Peptostreptococcus* were significantly lower [6]. The findings indicate that DMFT is related with the microbiota dysbiosis [20] and should be considered when researched the oral microbiota in SS patients. 

The other potential aspect that oral health status impacts the oral microbiota is the carriage of oral *Candida*. In one study, 87.5% of hyposalivation patients were colonized by *Candida*, which was isolated from saliva, with the majority (80.6%) being *C. albicans* [21]. For SS patients lacking saliva-mediated prevention of *Candida* adhesion to the mucosa [22], oral signs of candidiasis were evident in 13.1% to 60% of SS patients [23,24], which was significantly higher than the rate of candidiasis in healthy controls [25]. During the past decade, there has been increased recognition of the synergistic or antagonistic interactions between oral *Candida* and the oral microbiota [26,27]. *C. albicans* are widely distributed within the architecture of caries biofilms and might influence the ecology of cross-kingdom microbiota in caries [28]. Therefore, the carriage of *Candida* should also be considered in studies of the oral microbiota in SS patients.

This study explored the differences in the salivary microbiota in SS patients positive for *Candida*, with DMFT comparable to a control group of non-SS patients. This comparison has not been explored previously to our knowledge. Next-generation sequencing and Illumina-based sequencing of bacterial 16S rRNA were used to analyze microbiota diversity using unstimulated whole saliva rinse samples from *Candida* carriage SS patients and age-, gender-, and DMFT-matched control individuals.

## 2. Materials and Methods

### 2.1. Participants 

Because pSS has a strong female propensity, all participants in this study were female [29]. They were recruited at Peking University School and Hospital of Stomatology, Beijing, China, from January to August 2017 consecutively. The individuals in the Test group were diagnosed with primary SS according to the revised international classification criteria proposed by the American–European Consensus Group, 2002 [30]. They also met the 2016 ACR-EULAR Classification Criteria for primary Sjögren’s Syndrome, with a total score ≥4 [31]. All participants were interviewed. Those who had not received antibiotics in the preceding 3 months and had no systemic diseases (including hepatitis, tuberculosis, and diabetes) and no smoking or alcohol abuse were enrolled. All enrolled participants provided signed informed consent. This study was approved by the Institutional Review Board of Peking University School and Hospital of Stomatology (#PKUSSIRB-201947088). 

### 2.2. Clinical Examinations 

A comprehensive oral examination was performed by an experienced dentist through visual/tactile methods after air drying of the mouth. The total numbers of teeth present, decayed (D), missed (M), filled (F) teeth (T)/surfaces (S), incisal/cusp caries, and root surfaces caries were recorded separately. The dental examination was completed within 15 min, and the findings were recorded by an assistant. The examiner checked the record after the examination. Individuals with more than 20 teeth were enrolled. To compare the oral health status, the inclusion criteria for SS patients (Test group) were DMFS ≥15, teeth with active caries ≥5, and unstimulated whole salivary flow rate (UWSF) <0.1 mL/min. The respective values for oral candidiasis patients (positive control group, Group P) and healthy control group (Group N) were ≥10, ≥1, and ≥0.1 mL/min. Patients with severe chronic periodontitis (probing depth ≥ 6 mm) were excluded. Concentrated oral rinse fluid was cultured on Sabouraud dextrose agar. *Candida* counts >200 colony-forming units (CFU)/mL indicated *Candida* carriage for SS and candidiasis patients. Finally, the total of 15 patients included 5 *Candida* carriage SS patients in the Test group (A01 to A05), 5 patients in Group P (B01 to B05), and 5 caries active healthy patients in Group N (C01 to C05). Details of the original data from clinical examination was uploaded as Supplementary Materials.

### 2.3. Whole Saliva Collection

All included patients stopped oral hygiene 24 h prior to sample collection and did not eat on the morning of the examination day. The participants were instructed to rinse their mouths thoroughly for 1 min with 10 mL of sterile saline. The saline was completely spit into a sterile graduated test tube. Each tube was stored at −80 °C until analyzed.

### 2.4. DNA Isolation, PCR Amplification, and High-Throughput Sequencing 

All samples were washed twice with TE buffer (10 mM Tris-HCl, 1M EDTA, pH 8.0). Bacterial DNA was isolated and purified using a Wizard Genomic DNA Purification Kit (Promega, Madison, WI, USA) according to the manufacturer’s instructions. The concentration of the purified DNA was determined using a NanoDrop 2000 spectrophotometer (Thermo Fisher Scientific, Waltham, MA, USA) at an optical density of 260 nm (OD260). DNA integrity was verified by 1% agarose gel electrophoresis. For each individual sample, a standard concentration of 10 ng/uL DNA was diluted for the subsequent PCR assay and then stored at −80 °C.

High-throughput sequencing of all samples was performed by Cnkingbio Lab (Beijing, China). The V3−V4 hypervariable regions of the bacterial 16S rRNA gene were amplified by PCR using a GeneAmp 9700 device (Applied Biosystems, Foster City, CA, USA) from the isolated DNA, with primers 515F (GTGCCAGCMGCCGCGGTAA) and 806R (GGACTACHVGGGTWTCTAAT) and a unique barcode. PCR involved 10 min at 95 °C, followed by 35 cycles of 10 s at 95 °C, 30 s at 55 °C, and 30 s at 72 °C, and a final 10 min extension at 72 °C. The amplified PCR products were purified and checked with 2% agarose gel electrophoresis. The final amplicon preparation products were pooled in an emulsion PCR performed using a GS Lib-L kit (Roche Diagnostics GmbH, Mannheim, Germany) at a molecules-per-bead ratio of 0.7 according to the manufacturer’s instructions. Sequencing was performed on a HiSeq 2500 system (Illumina, San Diego, CA, USA) using paired-end and dual-end sequencing for the V3−V4 region.

### 2.5. Data Processing and Statistical Analyses

The generated sequences from Illumina HiSeq 2500 system were annotated as unique target sequences (raw tags) by FLASH (V1.2.7, http://ccb.jhu.edu/software/FLASH accessed on 9 October 2017). Clean tags were generated by filter raw tags using QIIME quantity control process (V1.9.0, http://qiime.org/scripts/split_libraries_fastq.html, accessed on 9 October 2017) [32]. The quality filtering strategy was performed by tags truncation and tags length. The effective tags sequences were selected by redundancy removal to remove chimera and impurity sequences using Mothur software v1.35.1 (http://www.mothur.org/wiki/Download_mothur, accessed on 9 October 2017) [33]. The effective unique tag sequences pre-clustered using the single linkage pre-clustering approach with 97% similarity were defined as one operational taxonomic unit (OTU). Species annotation of OTUs were classified to study the species composition diversity of the samples. The representative sequences from each OTU were compared and aligned to the SILVA database (Version 1123, http://www.mothur.org/wiki/Silva_reference_files, Bremen, Germany, accessed on 9 October 2017). 

Average age was compared by independent-samples t-test. Clinical data (DMFT, DMFS, incisal caries) were compared by the chi-square test in the three groups via SPSS 20.0 (IBM, Armonk, NY, USA). OTU differences across groups were analyzed by Metastat analysis using Mothur software v1.35.1 (http://www.mothur.org/wiki/Download_mothur, accessed on 9 October 2017). Alpha diversity was defined as the mean species diversity in sites (within-community) or habitats at a more local scale. Alpha diversity is the number of bacterial taxa and the proportional representation of each taxon per sample. The ACE, Chao, Shannon, and Simpson indices of alpha diversity were tested using the Wilcoxon rank-sum test. To examine the differences in the alpha diversity, data were analyzed using Bayesian methods by the limma package in R software (v2.2.0). Beta diversity was determined by principal component analysis (PCoA) based on weighted Unifrac matrix using R software (v2.2.0). Analysis of similarities (ANOSIM) determined the difference in bacterial composition and structure among the three groups. 

## 3. Results

### 3.1. Patients Characteristics

The baseline characteristics of SS patients and controls are presented in Table 1. With 15 participants, significant differences were not evident for the mean age, DMFT, and DMFS (*p* > 0.05) among the three groups (Table 1). Incisal caries was examined only in the Test group of SS patients.

### 3.2. General Outline

**Figure 1:**
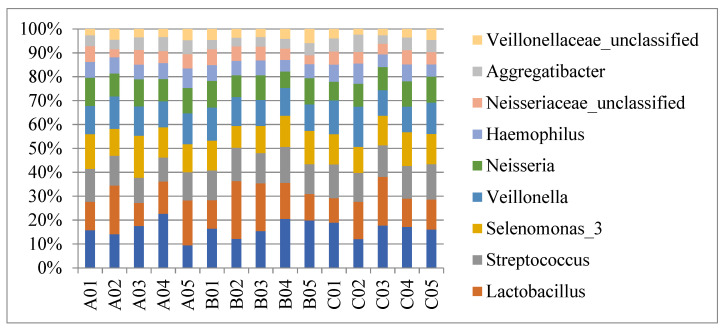
Species profiling histogram showing the 10 most predominant genera of saliva in Test group (A01–A05)/Group P (B01–B05)/Group N (C01–C05). The abundance of the total sequences of the five most predominant genera in Test group/Group P/Group N were *Treponema* (10.2%/9.9%/10.2%), *Lactobacillus* (9.5%/10%/8.4%), *Streptococcus* (7.6%/7.9%/8.5%), *Selenomonas* (8.6%/7.1%/7.8%), and *Veillonella* (7.9%/7.1%/7.8%). The numbers and proportions of sequences identified at the genus level were comparable among the three groups (*p* > 0.05).

An average of 132,153 (ranging from 13,112 to 298,785) total pairs reads was obtained. An average of 129,221 combined reads as effective tags was obtained after splicing using Flash software. A total of 3399 OTUs, comprising 1016 in the Test group, 1298 in Group P, and 1083 in Group N, were obtained from 15 samples. The samples comprised 10 phyla, 22 classes, 43 orders, 67 families, and 121 genera. The 121 genera comprised 85 genera in the Test group, 101 in Group P, and 95 in Group N. The ten most predominant genera constituting the sequences detected in saliva are presented in Figure 1. The five most predominant genera in the Test group/Group P/Group N were *Treponema* (10.2%/9.9%/10.2%), *Lactobacillus* (9.5%/10%/8.4%), *Streptococcus* (7.6%/7.9%/8.5%), *Selenomonas* (8.6%/7.1%/7.8%), and *Veillonella* (7.9%/7.1%/7.8%). The numbers and proportions of sequences identified at the genus level were comparable among the three groups (*p* > 0.05).

**Figure 2:**
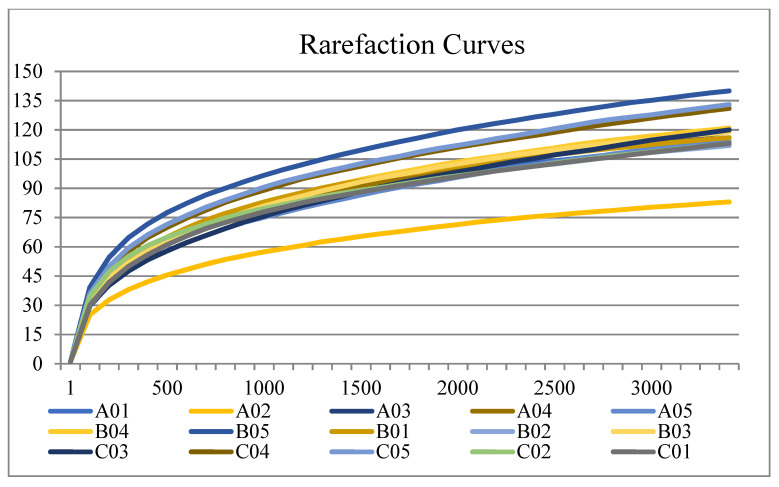
Rarefaction curves showing that sequencing reached saturation.

**Figure 3:**
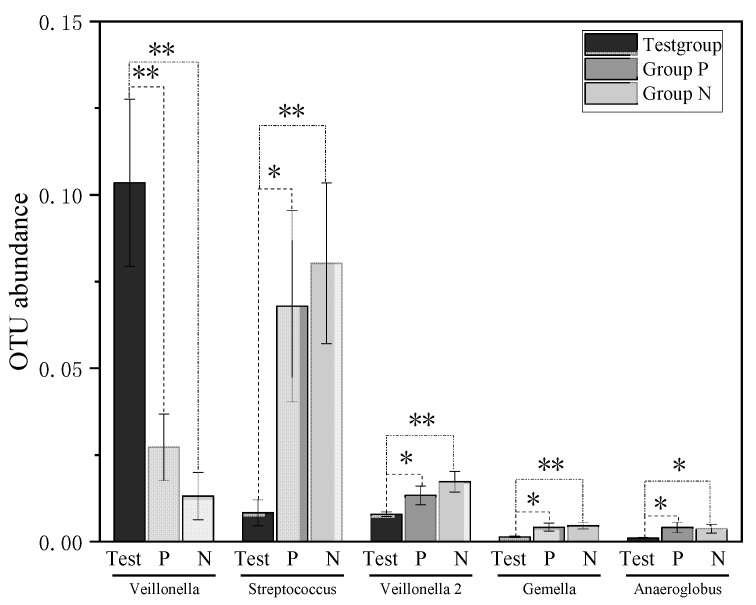
Five OTUs with mean abundance of taxonomy >0.001, displayed significant changes among all three groups: OTU001, genera *Veillonella*; OTU011, genera *Streptococcus*; OTU021, genera *Veillonella*; OTU045, genera *Gemella*; and OTU047, genera *Anaeroglobus*. * significantly different (*p* < 0.05); ** extremely significantly different (*p* < 0.01).

Rarefaction curves of samples are presented in Figure 2. Significant differences (*p* < 0.05) in the abundance of the taxa from saliva was estimated at the species level using Metastat analysis. Compared with Group P, 19 OTUs were significantly different in the Test group, with 7 OTUs increased and 12 OTUs decreased. Compared with Group N, 26 OTUs were significantly different in the Test group, with only 1 OTU (OTU0001, *Veillonella parvula*) increased and 25 OTUs decreased. Low taxonomy abundance is defined as an abundance less than 1% [34]. More details about taxonomy abundance greater than 0.001 are presented in Figure 3 and Table 2.

### 3.3. Biodiversity of the Salivary Microbiota

**Figure 4:**
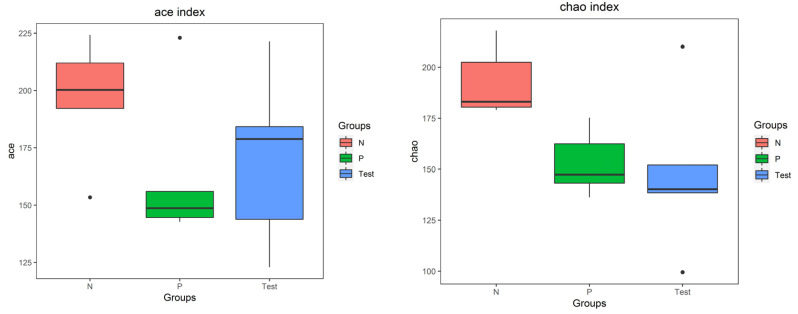

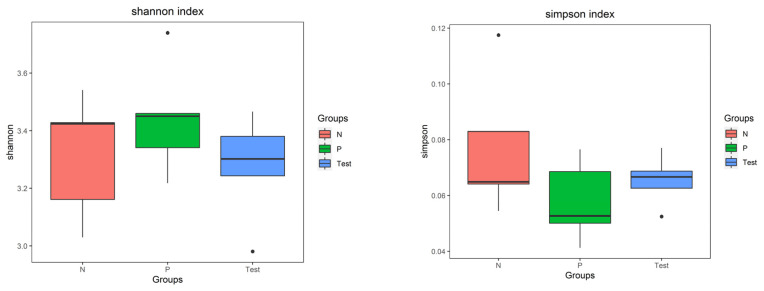
Alpha diversity (Ace, Chao, Shannon, and Simpson indices) of the three groups at the genus level. Microbiota community richness was analyzed by the ACE and Chao indices. Community diversity was analyzed by the Shannon and Simpson indices. Box plots indicate medium, minimum, and maximum values. Significant differences among three groups were evident for the four indices (*p* < 0.05).

**Figure 5:**
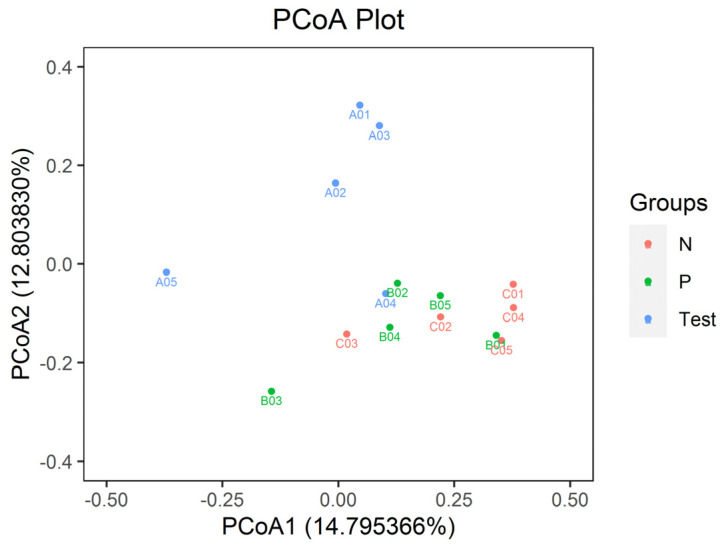
Beta diversity evident in principal coordinate analysis (PCoA) based on weighted Unifrac matrix. Similarity in bacterial composition is indicated by the distance between dots, with smaller distance denoting increased similarity. The spatial distribution of plots from Group P and N subjects overlapped, suggesting a similar set of genera. Plots from the Test group of subjects formed a cluster, having roughly greater values than the plot clusters from Group P and N subjects. Test group samples displayed a trend of dispersion along the PCoA2 axis. Group P and N samples displayed a trend of dispersion along the PCoA1 axis.

For alpha diversity, microbiota community richness was analyzed by the ACE and Chao indices, and the community diversity was analyzed by the Shannon and Simpson indices. For all four indices, significant differences were evident among the three groups (*p* < 0.05), as in Figure 4. Results of the beta diversity of the salivary microbiota determined by PCoA is shown in Figure 5. A clear separation of sample distribution was evident in the Test group, with roughly greater values than those from both Group P and Group N (principal components of 12.80% and 14.80%, respectively).

ANOSIM analysis also revealed significant differences in bacterial composition and structure among three groups (R = 0.304889, *p* = 0.005 < 0.01, respectively). Significant differences were evident in bacterial composition and structure between the Test group and Group P (R = 0.308, P = 0.043 < 0.05, respectively) and the Test group and Group N (R = 0.612, P = 0.003 < 0.01, respectively). No significant differences in bacterial composition and structure were evident between Group P and Group N (R = −0.032, *p* = 0.551 > 0.05, respectively).

## 4. Discussion

Our study findings strongly support the existence of distinct differences in the composition of the salivary microbiota in SS patients, independent of *Candida* carriage and DMFT. These findings further suggest that dysbiosis in oral microbiota might be a characteristic in SS patients. 

*Treponema*, *Lactobacillus*, *Streptococcus*, *Selenomonas*, and *Veillonella* were the dominant bacterial genera in saliva. *Treponema*, especially *T. denticola*, is commonly associated with periodontal disease [35]. *Lactobacillus*, *Streptococcus*, *Selenomonas*, and *Veillonella* have been significantly associated with the progression of dental caries [20]. *Lactobacillus* and *Streptococcus* are well-known cariogenic bacteria. Yet, prior studies have reported discordant results. One study found a similar salivary abundance of *Lactobacillus* and *Streptococcus* compared to a healthy population [6]. In contrast, a higher abundance in SS patients than a healthy population was found for *Lactobacillus* in two studies [17], and a similar abundance for *Streptococcus* also in two studies [14] but low in another study [36]. The reported abundance of *Selenomonas* increased in three studies [6,19,37] but was low in another [36]. Another predominant bacterial genera, *Prevotella* and *Veillonella*, was enriched in salivary microbiota in two studies [6,14]. The abundance of *Neisseria* was decreased in two studies [16,38]. 

In this current study, we did not observed differences in the abundance of any of these taxa. There are two possible explanations for this discrepancy. One is dental caries severity caused by oral cariogenic bacteria, and the other is the interaction between oral *Candida* and oral bacteria. Dental caries is a bacterial disease caused by dysbiosis in the complex microbiota community [21]. SS patients exhibit higher DMFT and DMFS. Our results agreed with the prior observations that with comparable DMFT, the salivary microbiota with most abundant genera of *Prevotella, Veillonella,* and *Streptococcus* in SS patients did not differ significantly from healthy controls [18]. Moreover, the relative abundance of *Veillonella* in SS patients with higher DMFT was reported to be higher than the abundance in healthy controls [6]. In this current study, the nonsignificant differences in the abundance of these cariogenic bacterial genera among the groups could be attributed to the comparable DMFT, leading to similar microbiota community dysbiosis.

*Candida* is a major cause of the opportunistic disease. The higher prevalence of oral *Candida* has been associated with hyposalivation and adverse effects on oral health [39]. Increasing evidence is solidifying the interaction between oral *Candida* and oral cariogenic bacteria, especially *C. albicans* [40]. In early in vitro oral biofilms, the presence of *C. albicans* alters the bacterial microbiota, leading to the presence of strictly anaerobic bacteria when oxygen is abundant [26]. The presence of *C. albicans* would increase the abundance of *Streptococcus* (particularly *S. mutans*), certain *Lactobacillus* species, and salivary *Veillonella* and *Prevotella* [26,41] and also increase the cariogenic virulence of biofilms [42] and even serve as potential “keystone” components of oral biofilms [43]. Oral *Candida* are also correlated with the number of untreated DMFT [44]. It was reported that *Candida*-infected individuals had 2.3 times more dental caries than those without *Candida* in their oral cavity [45]. *C. albicans* promotes caries activity that is dependent on PHR2 in a mixed microbial consortium [46]. In this current study, with similar oral *Candida* carriage and comparable DMFT, the abundance of the salivary microbiota at the genus level was not significantly different between SS patients with controls.

At the species level, the significantly increasing abundance of *V. parvula* (OTU001) in SS patients than controls was observed in this present study. The presence of *Veillonella* in the salivary microbiota has been related to the occurrence of dental caries [47]. *Veillonella* is a gram-negative anaerobic coccus capable of fermenting organic acids such as malate and lactate. This may result in increasing abundance of the salivary microbiota in SS patients [48]. The survival of *V. parvula* can alter the physiology of *S. mutans* by changing the expression of genes coding for many proteins [49]. This could contribute to more prevalent carious lesions in SS subjects. In this current study, even with comparable DMFT, significantly more *V. parvula* was detected in SS patients. These findings demonstrate the connection between *V. parvula* and the autoimmune disease SS. Elevated dysbiosis of *V. parvula* can initiate the progression of SS [50]. Hence, *V. parvula*, as an immunomodulatory commensal bacterium, may serve as a unique microbial biomarker for SS. This suggestion is supported by the results in other studies [6,14,51]. Uniquely, this present study is the first confirmation of the dominant role of *V. parvula*. Detection of the abundance of *V. parvula* might benefit the diagnosis, treatment, and monitoring of SS. 

Measurement of microbial community diversity can be critical for understanding the composition of the microbiota [52]. Different from previous studies [16,17,19,38], alpha diversity analyses of each sample revealed significantly higher diversity and more equal distribution of abundance in samples from SS patients than from control patients in this present study. Our results are supported by prior studies [6,14,53], which also reported higher alpha diversity in SS patients compared to healthy controls. Concerning beta diversity, the differentiation among sites or habitats contrasts with previous study findings [54]. In this present study, we observed more diverse bacterial composition of the saliva microbiota from SS patients. Similar results were previously reported in SS patients compared to a control group [36]. Normally, the difference in the oral microbial diversity of patients might be related to xerogenic medications, oral health status, saliva flow rates, salivary pH, severity of dental caries, and oral *Candida* carriage. Compared with oral *Candida* carriage and dental caries severity, the significant differences in microbial diversity might be a result of the effects caused by SS on the oral environment. ANOSIM analysis indicated SS itself might be associated with oral microbial dysbiosis. This was also a conclusion previously [14]. 

Our collective results suggest that SS patients with *Candida* carriage have different diversity and abundance of microbiota composition compared to patients with only *Candida* carriage and healthy patients, irrespective of DMFT. It remains unclear whether these changes observed in microbiota are the cause or consequence of SS. Interaction between the oral microbiota and autoimmune diseases is complex. For SS, activated CD4+ T cells and B cells infiltrate into the salivary glands [55]. Salivary gland destruction changes in saliva protein composition, which leads to microbiota dysbiosis in the oral cavity [50]. Changes in the oral microbiota may also be a factor for the onset of systemic autoimmune diseases, including SS [50]. Autoimmune diseases might be influenced by oral microbiota through the activation of Toll-like receptors, molecular mimicry, epitope spread, and antigen persistence [56]. The molecular mimicry theory posits that immune cells respond to self-antigens sharing epitopes with foreign antigens from the microbiota. Animal studies have demonstrated that peptides from oral bacteria could activate Ro60-reactive T cells [57], followed by the activation of B cells to plasma cells that produce SS antigen A [56]. Future studies should examine interactions between microbiota imbalances and autoimmune responses in the development and progression of SS, especially the role of *V. parvula*.

The diagnosis and management of SS represent a challenge for the clinician, especially the dentist. Accompanying various lesions and pseudoconditions of the oral mucosa, the differential diagnosis must be made [58]. This study found significant dysbiosis in the oral microbiota of SS patients through the marked change in salivary microbiota compared with controls. This study provided a potential way to help with diagnosis and management. There are some limitations of this study. First, the sample size is small, with only five in each group. This may have limited the power to detect significant associations. However, for the high-throughput sequencing based experiments, power and sample size calculations are not established. In some oral microbiome-related references, comparisons have involved 11 subjects divided into 2 groups [59] and 10 SS patients divided into 2 subgroups [60]. These two studies attained reasonable results and conclusions. Therefore, the five subjects for each group in this study were likely sufficient to prove the composition diversity and identify the most important bacteria in samples [61]. Second, 16S rRNA gene sequencing remains the gold standard of environmental microbiology. However, species level classification is not reliable with 200–400 bp reads of the 16S rRNA gene, and analyses have been confined to genus and higher taxonomies [62]. Finally, as one of the clinical characteristics, the UWSF in primary SS patients was −0.43 mL/min compared to controls [63]. It is difficult to conclude whether the microbial dysbiosis was caused by SS or hyposalivation. Future studies should involve many more patients (females and males) and controls to provide a more comprehensive view of microbiota composition. Quantitative real-time PCR should be used with standard strains to determine the absolute abundance of each taxon.

## 5. Conclusions

Oral saliva microbiota changes are documented for the first time in oral *Candida* carriage SS patients compared to control patients of comparable age and DMFT. The present findings extended the depth of previous findings. *V. parvula* might be a unique immunomodulatory commensal bacterium and perhaps a microbial biomarker for SS. The abundance of dominant bacteria did not differ significantly between the groups. However, the distinct community structure diversity identified microbial dysbiosis as a significant characteristic in SS patients, irrespective of oral *Candida* carriage and dental caries status.

## Figures and Tables

**Table 1:** Demographic characteristics of the three groups of patients.

	Test Group	Group P	Group N	*p*-Value
Number of cases	5	5	5	NA
Mean age (years) ^#^	59.8 ± 7.5	57.4 ± 15.2	45.0 ± 9.4	0.122
Women (n, %)	5 (100%)	5 (100%)	5 (100%)	NA
Smoker (n)	0	0	0	NA
Autoimmune diseases other than pSS (n)	0	0	0	NA
Received DMARDs in last 3 months (n)	3	0	0	NA
Duration of clinically apparent xerostomia (median months, range)	24 (6–36)	0 (0–0)	0 (0–0)	NA
Ocular dryness (n)	3	0	0	NA
Positivity of anti-SSA (n)	3	0	0	NA
LSG biopsy focus score ≥ 1(n)	2	0	0	NA
*Candida* carriage (cfu/mL)	>200	>200	0	NA
Wearing removable denture (n)	1	1	0	NA
DMFT ^#^	21.6 ± 6.7	17.4 ± 6.5	13.6 ± 3.8	0.137
DMFS ^#^	67.6 ± 12.9	58.0 ± 27.9	34.0 ± 19.7	0.069
Total incisal caries UWSF (mL/min)	3 0	0 >0.1	0 >0.1	0.148 0.047 *

Test group: SS patients group; Group P: oral candidiasis patients (positive control group) and Group N: healthy control patients. ^#^ Values are presented as mean ± standard deviation. * A *p* < 0.05 was considered statistically significant. DMARDs disease modifying anti-rheumatic drugs, LSG labial salivary gland, DMFT/DMFS total numbers of teeth present decayed (D), missed (M), filled (F) teeth (T)/surfaces (S), UWSF unstimulated whole salivary flow rate, NA not applicable.

**Table 2:** (**a**) Different taxonomy between Test group and Group P, with mean abundance > 0.001. (**b**) Different taxonomy between Test group and group N, with mean abundance > 0.001.

(a)				
OTU	Taxonomy (Genera)	Mean ± SD Test Group	Mean ± SD Group P	Metastat *p*-Value
Increase in SS				
OTU001	*Veillonella*	0.103472 ± 0.024092	0.027183 ± 0.00953	0.007 **
OTU038	*Gemella*	0.01297 ± 0.002196	0.006591 ± 0.000599	0.008 **
OTU042	*Neisseria*	0.019715 ± 0.008202	0.002831 ± 0.000635	0.039 *
OTU066	*Lactobacillus*	0.005269 ± 0.001321	0.00035 ± 0.0003	0.002 **
OTU102	*Selenomonas*	0.001091 ± 0.000422	0.000218 ± 0.000096	0.042 *
Decrease in SS				
OTU008	*Neisseria*	0.017735 ± 0.007067	0.053774 ± 0.015188	0.030 *
OTU011	*Streptococcus*	0.008269 ± 0.003757	0.067866 ± 0.027617	0.032 *
OTU013	*Firmicutes*	0.003479 ± 0.001823	0.021472 ± 0.00627	0.009 **
OTU021	*Veillonella*	0.007866 ± 0.000652	0.013299 ± 0.002685	0.049 *
OTU045	*Gemella*	0.001301 ± 0.000211	0.004119 ± 0.001153	0.019 *
OTU047	*Anaeroglobus*	0.000994 ± 0.00015	0.004037 ± 0.001439	0.035 *
OTU060	*Cardiobacterium*	0.000349 ± 0.000087	0.005424 ± 0.002308	0.029 *
**(b)**				
**OTU**	**Taxonomy (Genera)**	**Mean ± SD** **Test Group**	**Mean ± SD** **Group N**	**Metastat** ***p*-Value**
Increase in SS				
OTU001	*Veillonella*	0.103472 ± 0.024092	0.013111 ± 0.006831	0.002 **
Decrease In SS				
OTU002	*Streptococcus*	0.082284 ± 0.006397	0.182442 ± 0.028316	0.003 **
OTU011	*Streptococcus*	0.008269 ± 0.003757	0.080205 ± 0.023212	0.006 **
OTU012	*Veillonella*	0.001973 ± 0.000453	0.006733 ± 0.001754	0.014 *
OTU015	*Neisseriaceae_unclassified*	0.011237 ± 0.002864	0.03873 ± 0.010131	0.015 *
OTU021	*Veillonella*	0.007866 ± 0.000652	0.017235 ± 0.002976	0.006 **
OTU045	*Gemella*	0.001301 ± 0.000211	0.004557 ± 0.00096	0.004 **
OTU047	*Anaeroglobus*	0.000994 ± 0.00015	0.003697 ± 0.001258	0.037 *
OTU050	*Megasphaera*	0.002128 ± 0.00047	0.007184 ± 0.002469	0.048 *
OTU058	*Selenomonas*	0.000647 ± 0.000179	0.01557 ± 0.005949	0.018 *
OTU061	*Veillonella*	0.001773 ± 0.000745	0.008027 ± 0.00244	0.020 *
OTU086	*Veillonella*	0.000267 ± 0.00011	0.003788 ± 0.001247	0.010 **

OTU, operational taxonomic unit; SS, Sjögren’s syndrome. * significantly different (*p* < 0.05); ** extremely significantly different (*p* < 0.01).

## Data Availability

The data presented in this study are available on request from the corresponding author.

## Supplementary Materials

The following supporting information can be downloaded at: https://www.mdpi.com/article/10.3390/jcm12041559/s1. It presented STROBE Statement and details data from the clinical examination.
